# Safety and effectiveness of FOLFOXIRI plus molecular target drug therapy for metastatic colorectal cancer: A multicenter retrospective study

**DOI:** 10.18632/oncotarget.26626

**Published:** 2019-02-01

**Authors:** Takatsugu Ogata, Hironaga Satake, Misato Ogata, Yukimasa Hatachi, Hayato Maruoka, Daisuke Yamashita, Hiroki Hashida, Madoka Hamada, Hisateru Yasui

**Affiliations:** ^1^ Department of Medical Oncology, Kobe City Medical Center General Hospital, Kobe, Hyogo, Japan; ^2^ Cancer Treatment Center, Kansai Medical University Hospital, Hirakata, Osaka, Japan; ^3^ Department of Clinical Laboratory, Kobe City Medical Center General Hospital, Kobe, Hyogo, Japan; ^4^ Department of Pathology, Kobe City Medical Center General Hospital, Kobe, Hyogo, Japan; ^5^ Department of Surgery, Kobe City Medical Center General Hospital, Kobe, Hyogo, Japan; ^6^ Department of Surgery, Kansai Medical University Hospital, Hirakata, Osaka, Japan

**Keywords:** metastatic colorectal cancer, FOLFOXIRI therapy, triplet, anti-EGFR antibodies, anti-VEGF antibodies

## Abstract

**Introduction:**

FOLFOXIRI plus bevacizumab has a promising efficacy as first-line systemic chemotherapy for metastatic colorectal cancer (mCRC). This study aimed to evaluate the safety and effectiveness of FOLFOXIRI plus antibodies.

**Results:**

Fifty-five patients were enrolled (median age: 60 years, males: 25, females: 30). Twenty-six subjects had RAS mutations and 29 had RAS wild-type. Anti-VEGF and anti-EGFR antibodies were administered to 38 and 17 patients, respectively. The most common severe adverse event was neutropenia (51%). The overall response rate (ORR) was 69% (55% with anti-VEGF antibodies and 100% with anti-EGFR antibodies; *P* = 0.190), and the disease control rate was 98% (stable disease: 16 patients). With a median follow-up period of 18.4 months, the median progression-free survival (mPFS) was 11.0 months and the median overall survival (mOS) was 41.9 months. The mPFS and mOS did not significantly differ between patients treated with anti-EGFR antibodies and those with anti-VEGF antibodies.

**Methods:**

We retrospectively collected data from mCRC patients treated with FOLFOXIRI plus antibodies between March 2014 and December 2017.

**Conclusions:**

FOLFOXIRI plus antibody therapy was effective in patients with mCRC. The response rate was higher in patients treated with anti-EGFR antibodies than in those treated with anti-VEGF antibodies.

## INTRODUCTION

The overall survival (OS) in metastatic colorectal cancer (mCRC) patients was approximately 30 months in several recent clinical trials [1–3]. The TRIBE study [3] found that FOLFOXIRI (infusional fluorouracil/ levofolinate/ irinotecan/ oxaliplatin) plus bevacizumab therapy is effective against mCRC. The FOLFOXIRI therapy has a promising antitumor activity. However, due to its toxic side effects, several clinical trials were conducted for dose optimization [4]. In addition, there are antibodies approved for mCRC treatment, the anti-vascular endothelial growth factor (VEGF) antibodies (bevacizumab, ramucirumab, aflibercept) and the anti-epidermal growth factor receptor (EGFR) antibodies (cetuximab, panitumumab) used in mCRC with wild-type *RAS* gene. Previous studies examining the efficacy of each antibody found that the antitumor activity, especially of the anti-EGFR antibody, was affected by the primary location of the tumor [5]. Therefore, the National Comprehensive Cancer Network guideline for mCRC indicates anti-EGFR antibodies only for left-sided colorectal cancer [6]. However, these findings were based on the doublet plus antibodies therapy, whereas FOLFOXIRI plus antibodies therapies have not been studied thoroughly. Although the efficacy of the FOLFOXIRI plus bevacizumab therapy was demonstrated in a phase III study, the TRIBE study [3], the efficacy of the FOLFOXIRI plus anti-EGFR antibody therapy was examined in several phase II studies, the MACBETH study [7], the TRIP study [8], and the VOLFI study [9]. Furthermore, there are only a few studies comparing the efficacy of FOLFOXIRI plus anti-EGFR antibody with FOLFOXIRI plus bevacizumab [10].

Moreover, the *BRAF V600E* status is recognized as the prognostic factor of mCRC [11], and the FOLFOXIRI plus bevacizumab therapy is recommended for mCRC patients with *BRAF* mutations [12]. However, there was only a few data on FOLFOXIRI plus anti-EGFR antibodies in mCRC patients with *BRAF* mutations.

This retrospective study aimed to evaluate the safety and effectiveness of the FOLFOXIRI therapy combined with antitumor antibodies as a first-line treatment.

## RESULTS

### Patient characteristics

Fifty-seven patients were treated with FOLFOXIRI plus molecular target drugs for mCRC. Two patients who did not receive FOLFOXIRI as first-line therapy were excluded from this study. The patients’ characteristics are summarized in Table 1. There were 25 male and 30 female patients (45% and 55%, respectively), and the median age at the time of treatment was 60 years (range, 33−74; IQR, 52−65). Anti-VEGF antibodies and anti-EGFR antibodies were used as molecular target drugs in 38 (69%) and 17 patients (31%), respectively. In the patients treated with anti-EGFR antibodies, the primary tumor location was on the left side in 14 and on the right side in 3 patients (25 and 5%, respectively). In the patients treated with anti-VEGF antibodies, the primary tumor location was on the left side in 26 and on the right side in 12 patients (47% and 22%, respectively).

**Table 1:** Patients’ characteristics

Characteristics	*n* = 55	%
Gender, male/ female	25/ 30	(45/ 55)
Median age, years (range)	60 (33–74)	not applicable
ECOG PS, 0/ 1/ 2	37/ 17/ 1	(67/ 31/ 2)
All *RAS*, wt/ mt	29/ 26	(53/ 47)
*BRAF*, wt/ mt/ unknown	42/ 3/ 10	(76/ 5/ 18)
*UGT1A1*, wt/ heterozygous SNP	28/ 26	(52/ 47)
Tumor location, right/ left	15/ 40	(27/ 73)
Primary resection, +/ −	37/ 18	(67/ 33)
Prior adjuvant therapy, +/ −	6/ 49	(11/ 89)
Liver metastasis, +/ −	31/ 24	(56/ 44)
Liver−limited metastasis, +/−	7/ 48	(13/ 87)
Lung metastasis, +/ −	23/ 32	(45/ 58)
Peritoneal metastasis, +/ −	15/ 40	(27/ 73)
Stage of diagnosis, II/ III/ IV	3/ 7/ 45	(5/ 18/ 82)
Antibodies, Bev/ Ram/ Cmab/ Pmab	34/ 4/ 14/ 3	(62/ 7/ 25/ 5)

Abbreviations: ECOG PS, Eastern Cooperative Oncology Group performance status; wt, wild type; mt, mutation; SNP, single nucleotide polymorphism; Bev, bevacizumab; Ram, ramcirumab; Cmab, cetuximab; Pmab, panitumumab.

The median number of cycles administered during induction phase or triplet therapy, which are provided in Table 2, did not significantly differ between anti-EGFR and anti-VEGF antibodies.

**Table 2:** The course of induction phase or triplet

Characteristics	Bev *n* =34	Ram *n* = 4	Cmab *n* = 14	Pmab *n* = 3	Total *n* = 55
Induction phase - number of cycles, median (range)	12 (2−32)	12 (10−14)	12 (1−26)	12 (3−12)	12 (1−32)
Triplet regimen - number of cycles, median (range)	8 (1−20)	12 (7−12)	7 (1−14)	12 (9−12)	8 (1-20)
PD during induction phase, +/− (%)	8/ 26	2/ 2	2/ 12	0/ 3	12/43 (22/78)
Conversion, +/− (%)	5/ 29	0/ 4	3/ 11	0/ 3	8/47 (15/85)

Abbreviations: PD during induction phase, progressive disease during induction phase; Bev, bevacizumab; Ram, ramucirumab; Cmab, cetuximab; Pmab, panitumumab.

### Response to the treatment

One patient could not be evaluated for the response and was excluded from the efficacy analysis because the treatment was stopped after the 1st cycle due to anaphylaxis induced by cetuximab. The overall response rate was 69% (complete response [CR], 7 patients; partial response [PR], 30 patients), and the disease control rate was 98% (stable disease [SD], 16 patients). A stratified analysis of the molecular target therapy regimens indicated that the response rate tended to be higher in patients receiving anti-EGFR antibodies than in those treated with anti-VEGF antibodies (Table 3A). Classified by sidedness and antibodies, the overall response rate is summarized in Table 3B, 3C.

**Table 3:** Best response stratified according to the antibodies

Antibodies	CR	PR	SD	PD	ORR	DCR
Anti-EGFR, *n* = 16^*^	2 (13%)	14 (88%)	0 (0%)	0 (0%)	100%	100%
Anti-VEGF, *n* = 38	5 (13%)	16 (42%)	16 (42%)	1 (3%)	55%	97%
*P* value	1.00	0.155	0.015	1.00	0.190	1.00

^*^:Treatment was stopped after the first cycle due to anaphylaxis induced by cetuximab and the patient was excluded from this analysis.

**Figure 1:**
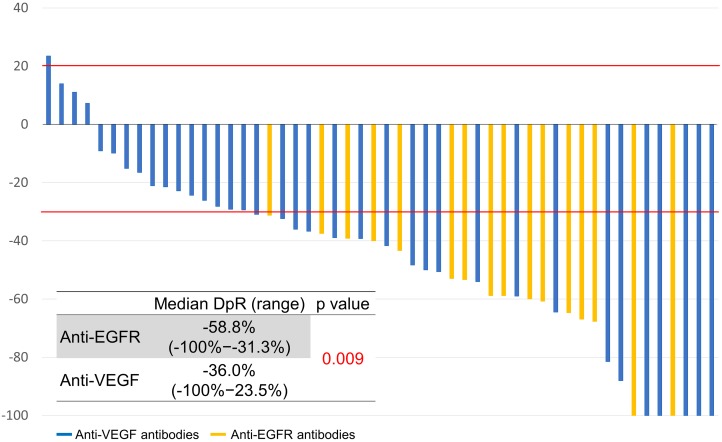
Depth of response

**Figure 2:**
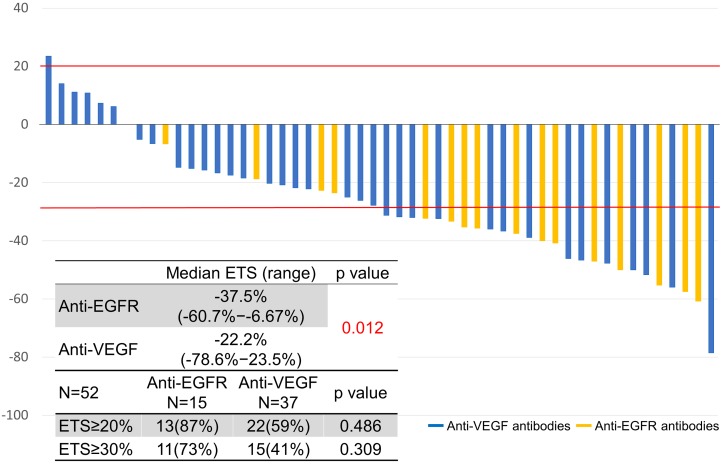
Early tumor response

The depth of response and early tumor shrinkage were assessed in 52 patients who had target lesions (Figures 1, 2). The depth of response was -41% (range, -100−24; IQR, -61−-28) and the early tumor shrinkage was -30% (range, -79−24; IQR, -40−-16) in all patients.

Classified by sidedness, the depth of response was -49% (range, -100−11; IQR, -61−-31) in left-sided tumors and -28% (range, -100−23; IQR, -59−-15) in right-sided tumors (*P* = 0.179). Early tumor shrinkage was -31% (range, -79−11; IQR, -45−19) in left-sided tumors and -20% (range, -57−23; IQR, -33−7) in right-sided tumors (*P* = 0.105).

Classified by sidedness in relation to the antibodies, the depth of response in left-sided tumors was -59% (range, -100−-31; IQR, -62−-50) with anti-EGFR antibodies and -39% (range, -100−23; IQR, -58−-28) with anti-VEGF antibodies (*P* = 0.062). The depth of response in right-sided tumors was -65% (range, -100−-40; IQR, -82−-52) with anti-EGFR antibodies and -23% (range, -100−23; IQR, -38−-4) with anti-VEGF antibodies (*P* = 0.101).

### Progression-free survival (PFS) and OS

**Figure 3:**
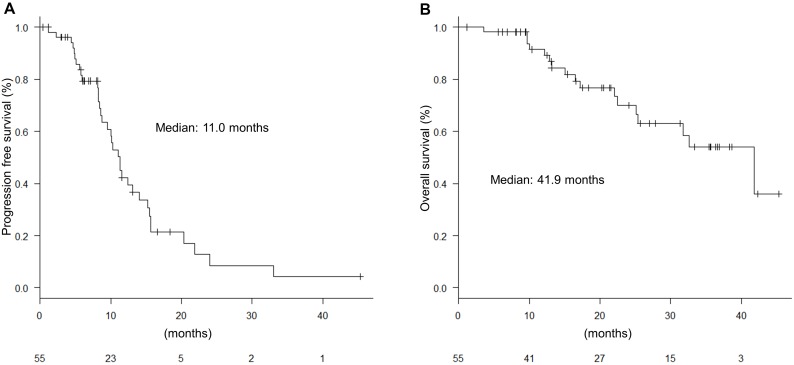
Progression-free survival (PFS) and overall survival (OS) (**A**) PFS for all patients; (**B**) OS for all patients.

**Figure 4:**
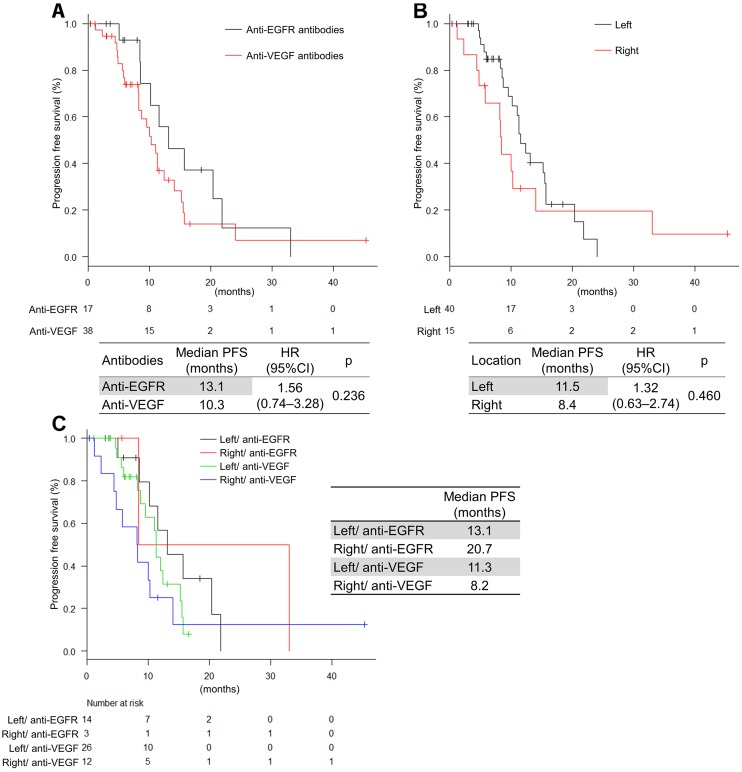
Progression-free survival by antibodies and tumor location (**A**) Classified by antibodies; (**B**) Classified by sidedness; (**C**) Classified by antibodies and sidedness.

With a median follow up of 18.4 months, the median PFS was 11.0 months (Figure 3A). The PFS did not significantly differ between anti-EGFR antibodies and anti-VEGF antibodies (13.1 months vs 10.3 months; hazard ratio [HR], 3.12 [0.88−11.0]; *P* = 0.143) (Figure 4A). The PFS was not significantly different in left-sided and right-sided tumors (11.5 months vs 8.4 months; HR, 1.32 [0.63−2.74]; *P* = 0.460) (Figure 4B). Multivariable analyses of PFS indicated that liver metastasis, peritoneal dissemination, and tumor location were associated with PFS (HR, 4.37 [1.80–10.6]; *P* = 0.001, HR, 0.27 [0.10–0.70]; *P* = 0.007, and HR, 4.23 [1.58–11.3]; *P* = 0.004, respectively) (Table 4A). If classified by primary location and antibodies, the FOLFOXIRI plus anti-EGFR antibodies tended to be more effective than the FOLFOXIRI plus anti-VEGF antibodies in left-sided primary tumors (Figure 4C).

Table 4Univariate and multivariate analyses of progression-free survival (PFS) and overall survival (OS)

**Table:** A) PFS

		Univariate			Multivariate		
		HR	95%CI	*P*	HR	95%CI	*P*
Age	<65/ ≥65	1.23	0.53–2.85	0.623			
*RAS*	mt/ wt	0.71	0.37–1.40	0.327			
*BRAF*	mt/ wt	2.79	0.80–9.68	0.093			
Liver meta	+/−	1.78	0.90–3.54	0.093	4.37	1.80–10.6	0.001
Lung meta	+/−	1.93	0.97–9.86	0.056			
Peritoneal meta	+/−	0.64	0.31–1.36	0.242	0.27	0.10–0.70	0.007
Tumor location	R/ L	1.32	0.63–2.74	0.460	4.23	1.58–11.3	0.004
Antibodies	VEGF/ EGFR	1.56	0.74–3.28	0.236

**Table:** B) OS

		Univariate			Multivariate		
		HR	95%CI	*P*	HR	95%CI	*P*
Age	<65/ ≥65	0.76	0.22–2.67	0.666			
*RAS*	mt/ wt	0.76	0.29–1.97	0.565			
*BRAF*	mt/ wt	5.82	1.55–21.8	0.003	13.1	2.37–72.9	0.003
Liver meta	+/−	5.57	1.59–19.5	0.003	5.16	1.15–23.0	0.031
Lung meta	+/−	1.20	0.46–3.12	0.704			
Peritoneal meta	+/−	1.19	0.44–3.23	0.729			
Conversion	+/−	0.21	0.03–1.61	0.098			
Tumor location	R/L	1.13	0.40–3.22	0.815			
Antibodies	VEGF/EGFR	2.65	0.75–9.30	0.115			
PD during induction phase	+/−	21.0	5.20–84.9	<0.001	12.5	2.71–57.8	0.001

Abbreviations: mt, mutation; wt, wild type; meta, metastasis; R, right; L, left; VEGF, anti- vascular endothelial growth factor antibodies; EGFR, anti-epidermal growth factor receptor antibodies; PD during induction phase, progressive disease during induction phase.

**Figure 5:**
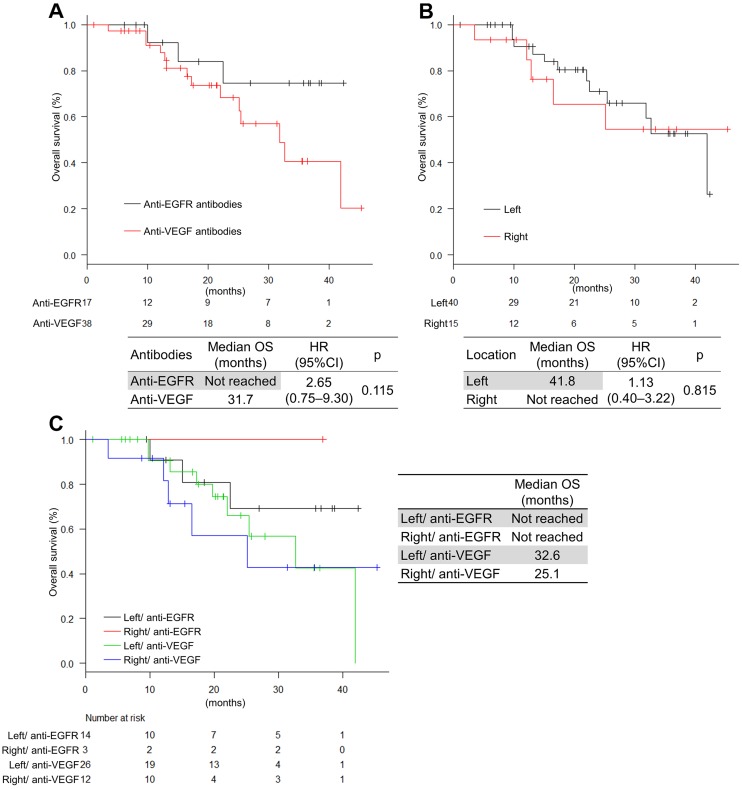
Overall survival by antibodies and tumor location (**A**) Classified by antibodies; (**B**) Classified by sidedness; (**C**) Classified by antibodies and sidedness.

The median OS was 41.9 months (Figure 3B). OS was not significantly different in the antibodies and the tumor location, respectively (not reached vs 36.7 months; HR, 2.65 [0.75−9.30]; *P* = 0.115 and 41.8 months vs not reached; HR, 1.13 [0.40–3.22]; *P* = 0.815, respectively) (Figure 5A, 5B). Multivariable analyses of OS showed that *BRAF* status, liver metastasis, and progressive disease (PD) during induction phase were associated with OS (HR, 13.1 [2.37–72.9]; *P* = 0.003, HR, 5.16 [1.15–23.0]; *P* = 0.031, and HR, 12.5 [2.71–57.8]; *P* = 0.004, respectively) (Table 4B). Moreover, if classified by primary location and antibodies, the FOLFOXIRI plus anti-EGFR antibodies tended to be more effective than the FOLFOXIRI plus anti-VEGF antibodies in left-sided primary tumors (Figure 5C).

### Conversion therapy

**Figure 6:**
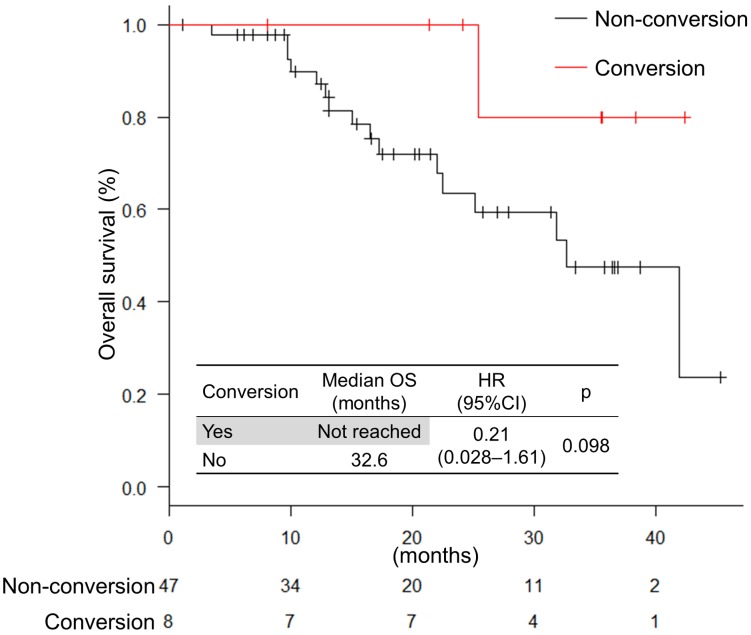
Overall survival in patients treated with conversion therapy

Eight patients (15%) received conversion therapy. The locations of the metastatic site in the patient subjected to conversion therapy were as follows: localized advanced, 1 patient; liver-limited, 4 patients; distant lymph node-limited, 1 patient; ovarian-limited, 1 patient; and liver and lymph node metastasis, 1 patient. Out of eight patients, five finished the conversion therapy with R0 resection and 3 with R1 resection. The response to the chemotherapy was classified as grade 1a, 2, and 3 (4 patients, 2 patients, and 2 patients, respectively). The median recurrence-free survival was 10.6 months (range, 2.6−24.0; IQR, 5.6−14.4). There was no significant difference in OS between conversion and non-conversion therapy (not reached vs 32.6 months; HR, 1.13 [0.40–3.22]; *P* = 0.815) (Figure 6). The number of patients who were converted to the surgery did not significantly differ between anti-EGFR and anti-VEGF antibodies (18% vs. 13%; *P* = 0.701).

At the date of analysis, 6 patients had recurrence and 2 patients with pathological response grade 3 had no recurrence. The sites of recurrence were as follows: distant lymph nodes, 2 patients; lungs, 2 patients; peritoneum, 1 patient; liver, 1 patient.

### Adverse events

**Figure 7:**
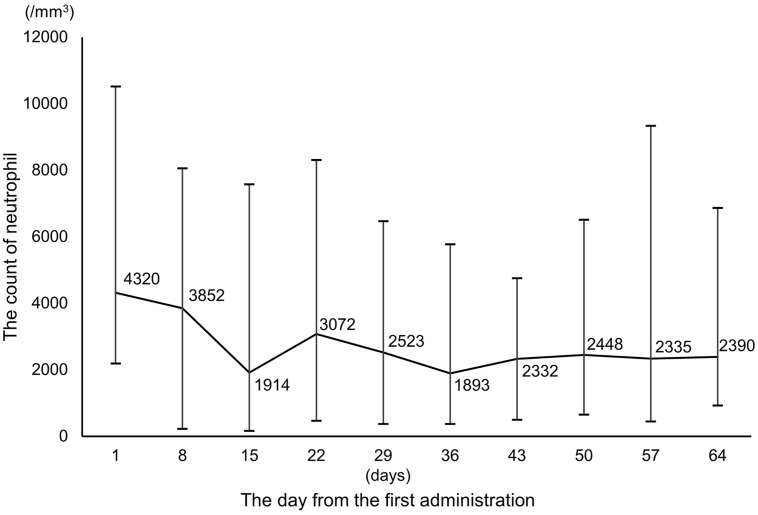
Changes in the neutrophil count during FOLFOXIRI plus molecular target drugs

The adverse events are summarized in Table 5. The most common adverse events of any grade were leukopenia and neutropenia (44 patients, 80%). The most common adverse events of grade 3 and 4 were neutropenia (28 patients, 51%). The median number of days until occurrence of the worst neutropenia was 39 days (range, 9–161 days) (Figure 7). Only 1 patient (2%) used granulocyte colony-stimulating factors.

Table 5Adverse events

**Table:** A) All patients (*n* = 55)

	Any grade, *n* (%)	Grade 3, *n* (%)	Grade 4, *n* (%)	Grade 3/4,%
Leukopenia	44 (80)	3 (6)	0	6
Neutropenia	44 (80)	20 (36)	8 (15)	51
Anemia	30 (54)	0	0	0
Thrombocytopenia	14 (26)	0	0	0
Hypomagnesemia	16 (30)	2 (4)	0	4
Alopecia	13 (24)	0	0	0
Fatigue	22 (41)	0	0	0
FN	0	0	0	0
Diarrhea	31 (57)	3 (6)	0	6
Anorexia	17 (32)	0	0	0
Nausea	29 (54)	1 (2)	0	2
Neurotoxicity	43 (81)	1 (2)	0	2
Hypertension	23 (43)	1 (2)	0	2
Proteinuria	27 (50)	1 (2)	0	2
Rash acneiform	18 (33)	0	0	0

**Table:** B) Classified according to antibodies (*n* = 55)

	Anti-EGFR (*n* = 17)		Anti-VEGF (*n* = 38)		
	Any grade, *n* (%)	Grade 3/4, *n* (%)	Any grade, *n* (%)	Grade 3/4, *n* (%)	*P* value
Leukopenia	14 (82)	1 (6)	30 (79)	2 (5)	1.00
Neutropenia	15 (88)	9 (53)	29 (76)	19 (50)	0.830
Anemia	6 (35)	0	24 (63)	0	0.318
Thrombocytopenia	6 (35)	0	8 (21)	0	0.527
Hypomagnesemia	10 (59)	2 (12)	6 (16)	0	0.039
Alopecia	4 (24)	0	2 (6)	0	0.169
Fatigue	6 (35)	0	16 (43)	0	1.00
FN	0	0	0	0	–
Diarrhea	13 (77)	2 (12)	18 (49)	1 (3)	0.350
Anorexia	8 (47)	0	9 (24)	0	0.253
Nausea	12 (70)	1 (6)	17 (46)	0	0.347
Neurotoxicity	13 (77)	0	30 (81)	1 (3)	1.00
Hypertension	2 (12)	0	21 (57)	0	0.045
Proteinuria	5 (29)	0	22 (60)	1 (3)	0.295
Rash acneiform	15 (88)	0	3 (8)	0	<0.001

**Table:** C) Classified by UGT1A1 status (*n* = 55)

	UGT1A1 wild type, *n* = 28		UGT1A1 single hetero, *n* = 26		
	Any grade, *n* (%)	Grade 3/4, *n* (%)	Any grade, *n* (%)	Grade 3/4, *n* (%)	*P* value
Leukopenia	21 (75)	0	23 (82)	3 (11)	0.839
Neutropenia	21 (75)	14 (50)	22 (85)	14 (54)	0.839
Diarrhea	19 (68)	2 (7)	12 (46)	1 (4)	0.498

**Table:** D) Classified by initial dose of irinotecan (*n* = 55)

	125 mg/m^2^, *n* = 8		150 mg/m^2^, *n* = 20		165 mg/m^2^, *n* = 25	
		Any grade, *n* (%)	Grade 3/4, *n* (%)	Any grade, *n* (%)	Grade 3/4, *n* (%)	Any grade, *n* (%)	Grade 3/4, *n* (%)
Leukopenia	7 (88)	0	16 (80)	1 (5)	21 (84)	2 (8)
Neutropenia	7 (88)	5 (63)	14 (70)	7 (35)	21 (84)	15 (60)
Diarrhea	5 (63)	0	13 (65)	0	12 (48)	3 (12)

Abbreviations: FN, febrile neutropenia; Anti-EGFR, anti-epidermal growth factor receptor antibodies; Anti-VEGF, anti- vascular endothelial growth factor antibodies.

### The regimens after disease progression

The regimens after disease progression are summarized in Table 6. Six patients with conversion therapy had recurrence and 35 patients had progression after treatment with FOLFOXIRI plus molecular target drugs. Five patients received triplet re-induction therapy (FOLFOXIRI plus molecular target agents). Fourteen patients received doublet therapy (irinotecan base, 10 patients and oxaliplatin base, 1 patient). Eleven patients received late-line therapy. Five patients received best supportive care.

**Table 6:** The regimen following disease progression

Regimen	*n* = 41	(%)
Triplet re-induction	5	12
Doublet + antibodies	14	34
Anti-EGFR ± CPT-11	6	15
Late-line	11	27
Best supportive care	5	12

Abbreviations: Anti-EGFR, anti-epidermal growth factor receptor antibodies; CPT-11, irinotecan.

### *BRAF V600E* mutation

The *BRAF* status was analyzed in 45 patients and 3 patients were positive for *BRAF V600E* mutation (Table 7). All patients were female, and the primary tumor location was on the right side in 2 patients. Two patients received bevacizumab and 1 patient received cetuximab. The median depth of response in patients with *BRAF V600E* mutation was -24% (range, -38 to 7). The median PFS in patients with *BRAF V600E* mutation was tended to be shorter than in those without (8.2 months vs 11.0 months; HR, 2.79 [0.80–9.68]; *P* = 0.093). The median OS in patients with *BRAF V600E* mutation was significantly shorter than in those without (12.9 months vs 41.9 months; HR, 5.82 [1.55–21.8]; *P* = 0.003).

**Table 7:** Patients with *BRAF V600E* mutation

	Sex	Age	Location	Antibody	DpR (%)	PFS (months)	OS (months)
Case 1	F	54	Left	Cmab	-38	5.1	10.0
Case 2	F	64	Right	Bmab	7	8.2	12.9
Case 3	F	52	Right	Bmab	-24	10.3	25.2

Abbreviations: F, female; Cmab, cetuximab; Bmab, bevacizumab; DpR, depth of response; PFS, progression free survival; OS, overall survival.

## DISCUSSION

In this study, the effectiveness of FOLFOXIRI plus molecular target drugs was very promising, which corroborates the findings of earlier studies. The FOLFOXIRI plus anti-EGFR antibodies had better results in tumor shrinkage than the FOLFOXIRI plus anti-VEGF antibodies.

### Differences in efficacy related to tumor location and antibodies

Recent reports found many differences in the clinical and molecular characteristics of colorectal cancers classified by tumor location [13, 14].

According to a sub-analysis of the CALGB/SWOG 80405 study [15], the median OS was significantly longer in left-sided than in right-sided tumors (33.3 months vs 19.4 months; *P* < 0.001). In patients with left-sided primary tumor, the median OS was significantly longer in patients treated with cetuximab than in those with bevacizumab (36.0 months vs 31.4 months; *P* = 0.018). In contrast, in patients with right-sided primary tumor, the median OS was tended to be shorter in patients treated with cetuximab than in those with bevacizumab (16.7 months vs 24.2 months; *P* = 0.065). A sub-analysis of the PEAK and the FIRE-3 study found the same tendency [5]. According to the meta-analysis of six trials (CRYSTAL study, FIRE-3 study, CALGB/SWOG 80405 study, PRIME study, PEAK study, and 20050181 study), the anti-EGFR antibodies were more effective in left-sided tumors than in right-sided tumors [5]. In our study, we also assessed the relationship between the primary tumor location and the antibodies.

In both tumor locations, anti-EGFR antibodies were associated with higher overall response rates as compared to those associated with anti-VEGF antibodies. Furthermore, the depth of response was also higher using anti-EGFR antibodies. However, the number of patients was too small to perform a combined analysis of primary locations and antibodies. A prospective study will be needed to support this type of analysis.

### Adverse events by antibodies

The profile of the adverse events differed by antibodies. Severe adverse events from previous studies are summarized in Table 8. Any grade diarrhea occurred more frequently in the patients treated with FOLFOXIRI plus anti-EGFR antibodies than in those without. In this study, severe diarrhea (≥Grade 3) also occurred in the patients treated with FOLFOXIRI plus anti-EGFR. Because we conducted a retrospective study, there is the possibility that adverse events were underestimated. Although the dosing of FOLFOXIRI plus anti EGFR antibodies is still being studied due to the toxicity, the TRIBE dose was used as the recommended dose in the JACCRO CC-14 study in Japan [16].

**Table 8:** Severe adverse events (≥Grade 3) in previous studies

%	TRIBE [3]	MACBETH [7]	TRIP [9]	VOLFI [22]
Neutropenia	50	31	48	16
Febrile Neutropenia	9	3	5	–
Diarrhea	19	18	35	25
Hypertension	5	–	–	–
Skin toxic effect	–	18	14	3
Hypomagnesemia	–	–	13	–

### Adverse events by *UGT1A1* status

UDP-glucuronosyl transferase (UGT) metabolizes SN-38, which is the active metabolite of irinotecan. UGT1A1^*^6 and UGT1A1^*^28 are polymorphisms of *UGT1A1*. Patients homozygous for ^*^6 or ^*^28 are reported to be associated with severe adverse events [17]. In this study, febrile neutropenia did not occur. In the QUATTRO study [18], a heterozygous single nucleotide polymorphism was also associated with high toxicity; however, this was a retrospective study and there was no significant difference. We believe, more research on homozygous polymorphisms of *UGT1A1* is needed. Regarding the use of modified FOLFOXIRI therapy, there was no difference in toxicity between non-homozygous and homozygous polymorphisms. Thus, modified FOLFOXIRI therapy can be possibly performed safely [4].

### Conversion therapy

The conversion therapy is a standard therapy for mCRC patients, especially with liver-limited metastasis. Fong *et al*. [19] reported 5-year survival rates of 37% in patients with liver metastasis from colorectal cancer who underwent liver resection, whereas patients with untreated liver metastases survived for only 5–14 months [20]. In the OLIVIA study [21], the response rate was higher for FOLFOXIRI plus bevacizumab than for doublet plus bevacizumab (80.5% vs. 61.5%; *P* = 0.061) and the R0 resection rate was significantly higher for FOLFOXIRI plus bevacizumab than for doublet plus bevacizumab (48.8% vs. 23.1%; *P* = 0.017). In the VOLFI study [22], the resection rate was significantly higher for FOLFOXIRI plus panitumumab than for FOLFOXIRI (33.3% vs. 12.1%; *P* = 0.029). In this study, the resection rate was 15%. In the patient with liver-limited metastases, the resection rate was 57% (4 out of 7 patients). The OS in patients with conversion therapy tended to be longer than that in patients with non-conversion therapy.

### Therapy after disease progression

It is difficult to select a subsequent therapy when disease progression occurs in first-line FOLFOXIRI regimen, because its key drugs, 5-FU, irinotecan, and oxaliplatin, are first-line therapeutics. In the case of disease progression during maintenance therapy, FOLFOXIRI, FOLFOX or FOLFIRI can be selected as re-induction therapy. However, in the case of disease progression during induction therapy, only the late-line therapies will be selected. The therapy after disease progression during induction therapy has a very poor prognosis factor. In this analysis, irinotecan-based chemotherapy was most frequently selected as the doublet therapy. The tendency was similar to that in the TRIBE or QUATTRO study.

### Patients with *BRAF V600E* mutation

Metastatic colorectal cancer with *BRAF V600E* mutation is rare but has a very poor prognosis. *BRAF* mutations are also found in melanoma or lung cancer. These cancers are recommended to be treated with *BRAF* inhibitor combined with *MEK* inhibitors. Recently, in mCRC, clinical trials with *BRAF* inhibitors and *MEK* inhibitors have been performed, for example, the BEACON and SWOG 1406 trial, but these trials were targeted to pretreated patients. There has been no trial in chemotherapy-naïve patients with *BRAF V600E* mutation. According to the sub-analysis of the TRIBE trial, the OS was significantly longer for the FOLFOXIRI plus bevacizumab among other therapies [23]. In the Pan-Asia ESMO Consensus guideline for colorectal cancer, FOLFOXIRI plus bevacizumab is recommended as first-line therapy in patients with *BRAF V600E* mutation. In this analysis, the *BRAF V600E* mutation was detected in only 3 patients, who had a poorer median OS, as reported in previous studies. Because the number of patients with *BRAF V600E* mutation was small in this study, we cannot make a suggestion for using either anti-VEGF antibodies or anti-EGFR antibodies in these patients. In the future, more patients with *BRAF V600E* mutations should be studied.

### Limitations of this study

This study was the retrospective study conducted at two centers. In the future, we need to consider a prospective study for comparing FOLFOXIRI plus bevacizumab with FOLFOXIRI plus anti-EGFR antibody. The JACCRO CC-13 study (UMIN000018217) is ongoing. The result of this study may provide a solution to this question.

## MATERIALS AND METHODS

### Patients

We performed a retrospective study in two centers, Kobe City Medical Center General Hospital and Kansai Medical University Hospital, from March 2014 to December 2017. The patients with mCRC had histologically diagnosed adenocarcinoma, and they were in recurrence after surgery or *de novo* stage IV. For the patients treated with adjuvant therapy, 6 months or more had passed since completion of chemotherapy. They were treated with FOLFOXIRI plus molecular target drugs as first-line therapy. The following data were collected: gender, age, *RAS/BRAF* status, *UGT1A1* status, tumor location, primary resection, prior adjuvant therapy, liver metastasis, lung metastasis, peritoneal dissemination, stage at diagnosis, the types of therapeutic antibodies as well as their efficacy and safety during the protocol treatment.

This retrospective analysis was approved by the Institutional Review Board of Kobe City Medical Center General Hospital and Kansai Medical University Hospital.

### Treatments

The FOLFOXIRI plus bevacizumab therapy was reported in the TRIBE study. The original dose and schedule of FOLFOXIRI consisted of a 60 minute infusion of irinotecan at a dose of 165 mg/m^2^, and a 120 minute infusion of oxaliplatin at a dose of 85 mg/m^2^ combined with a concomitant 120 minute infusion of leucovorin at a dose of 200 mg/m^2^, followed by a 48 hour continuous infusion of fluorouracil to a total dose of 3200 mg/m^2^, repeated every 2 weeks [3]. In this study, protocol treatment was defined as chemotherapy consisting of FOLFOXIRI plus molecular target drugs, including some cases treated with a modified dose of FOLFOXIRI according to the clinical trials and *UGT1A1* status (Table 9). The molecular target drugs were bevacizumab, ramucirumab, cetuximab and panitumumab. The molecular target drug doses are listed in Table 9.

Table 9Initial dose of FOLFOXIRI plus molecular target drugs in this study

**Table:** A) All patients

	Bev	Ram	Cmab	Pmab
Antibodies	5 (mg/kg)	8 (mg/kg)	250^*^ (mg/m^2^)	6 (mg/kg)
5-FU		4200/3200 (mg/m^2^)		
CPT-11		125/150/165 (mg/m^2^)		
L-OHP		85 (mg/m^2^)		

^*^ 400 mg/m^2^ at 1st dose

**Table:** B) The initial dose of irinotecan classified by UGT1A1 status

	Wild type, *n* = 28 (%)	Single hetero, *n* = 26 (%)	*P* value
125 (mg/m^2^)	4 (15)	4 (15)	1.00
150 (mg/m^2^)	11 (41)	9 (35)	1.00
165 (mg/m^2^)	12 (44)	13 (50)	0.812

Abbreviations: Bev, bevacizumab; Ram, ramucirumab; Cmab, cetuximab; Pmab, panitumumab; CPT-11, irinotecan; L-OHP, oxaliplatin.

Induction treatment consisted of irinotecan and/or oxaliplatin and fluorouracil, leucovorin with molecular target drugs. After induction therapy, maintenance treatment with fluorouracil, leucovorin, and molecular target drugs was continued until disease progression. When the tumor was shrinking, conversion therapy was recommended.

### Assessments

Tumors were assessed every 8 weeks by computed tomography until disease progression. Response and progression were assessed according to RECIST, version 1.1. To assess the *RAS* and *BRAF* status, DNA was extracted from archival tissue specimens from the primary tumor or metastasis. Early tumor shrinkage was defined as the relative change in the sum of longest diameters of RECIST target lesions at week 8 compared to baseline, while depth of response was defined as the percentage of tumor shrinkage, based on the diameters of target lesions according to RECIST, version 1.1 at the lowest point as compared with the baseline values.

Molecular target drugs were classified into two arms, the anti-EGFR antibodies [cetuximab and panitumumab] and the anti-VEGF antibodies [bevacizumab and ramucirumab]. To classify the sidedness of the primary tumor location, the cancers located in the cecum and the ascending and transverse colon were defined as right-sided colon cancers, whereas the cancers located in the descending and sigmoid colon, and the rectum were defined as left-sided colon cancers.

In the case of conversion therapy, the surgical specimens of each patient were pathologically evaluated to score the pathological response using Japan criteria for assigning a grade [24]. The grading was performed according to the proportion of the tumor affected by degeneration or necrosis. The grades according to the Japan criteria were as follows: grade 0, none of the tumor affected; grade 1a, <1/3 affected; grade 1b, ≥1/3 and <2/3 affected; grade 2, ≥2/3 affected; and grade 3, no residual tumor.

### Statistical analysis

The depth of response and the early tumor shrinkage were analyzed by the χ^2^ test. The analysis was performed on all patients and classified according to antibodies and *BRAF* status. PFS was defined as the time from date of the first administration of FOLFOXIRI to the time until the earlier of disease progression or death due to any cause. We defined the patients with conversion surgery as censored on the date of surgery. Overall survival (OS) was defined as the time from date of the first administration of FOLFOXIRI to the date of death due to any cause or last confirmation of survival. Patients who were still alive were censored at the last follow-up examination. Potential predictive factors were age, *RAS* status, *BRAF* status, metastatic site, tumor location, and antibodies; these were subjected to a univariate analysis using the Kaplan–Meier analysis model; the differences between groups were compared by log-rank test. Predictive factors with significance values of *P* < 0.10 in the univariate analysis were further subjected to a multivariate analysis, which was performed using the Cox proportional hazards model. Potential prognostic factors were age, *RAS* status, *BRAF* status, metastatic site, conversion therapy, tumor location, antibodies, and PD during induction phase; these were analyzed like the predictive factors. Analysis was performed using R.

## CONCLUSIONS

FOLFOXIRI plus molecular target therapy was effective in patients with mCRC. The response rate was significantly higher in patients receiving anti-EGFR antibodies than in those treated with anti-VEGF antibodies, although the former patients tended to have a higher incidence of skin toxicities and hypomagnesaemia.

## Abbreviations

CR: complete response
EGFR: epidermal growth factor receptor
mCRC: metastatic colorectal cancer
OS: overall survival
PD: progressive disease
PFS: progression-free survival
PR: partial response
SD: stable disease
VEGF: vascular endothelial growth factor.
